# Supplementary material for: Analysis of genetic requirements and nutrient availability for Staphylococcus aureus growth in cystic fibrosis sputum
Source: mBio. 2025 Apr 2;16(5):e00374-25. doi: 10.1128/mbio.00374-25 (PMC12077221; doi:10.1128/mbio.00374-25)
Supplement: Supplemental Material — Fig S1-S3; Tables S3-S5. [file mbio.00374-25-s0001.docx]

**Genetic and nutrient analysis of cystic fibrosis sputum as a growth milieu for *Staphylococcus aureus***

Lauren M. Shull, Daniel J. Wolter, Dillon E. Kunkle, Katherine A. Legg, David P. Giedroc, Eric P. Skaar, Lucas R. Hoffman, Michelle L. Reniere

**SUPPLEMENTAL MATERIAL**

**Figure S1. Tn-seq results including SCFM3. A)** UpSet plot showing the extent of overlap of hits between different CF sputum media and SCFM3. Hits were defined as genes that were statistically significantly enriched or depleted by at least 2-fold in sputum media or SCFM3 compared to TSB. Significance was determined using the resampling method in TRANSIT and *p*-values were adjusted for multiple comparisons using the Benjamini-Hochberg procedure. Hits that were enriched in one or more samples but depleted in one or more other samples were not counted as overlapping. **B)** Heatmap showing all significant genes by ANOVA, with TSB as the reference condition.

**Figure S2. Elements quantified by ICP-MS in CF sputum.** Each point represents a sputum sample from an individual with CF.

**Figure S3. The role of each cysteine importer in cysteine toxicity of ∆*cymR*.** Growth kinetics of bacterial strains in SCFM1 containing the indicated organic sulfur source: A) cysteine, B) GSH, C) equimolar cysteine and GSH, D) cysteine and minimal GSH. Data are the mean and standard deviation of four independent experiments.

**Table S1. Tn-seq analysis in each medium** (separate Excel file)

**Table S2. Tn-seq analysis in all media by ANOVA** (separate Excel file)

**Table S3. Strains used in this study**

**Table S4. Plasmids and phages used in this study**

**Table S5. Oligonucleotides used in this study**


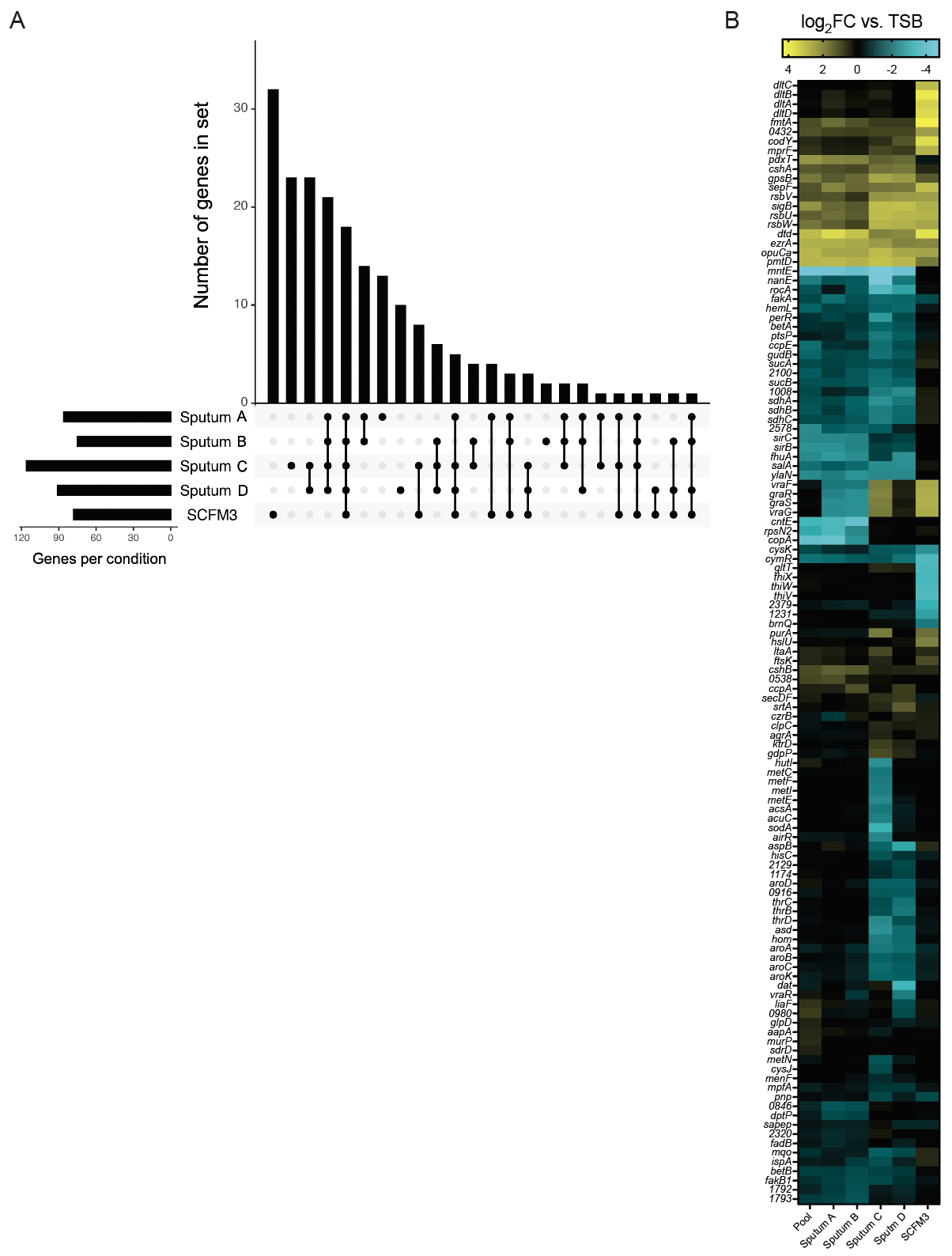


**Figure S1. Tn-seq results including SCFM3. A)** UpSet plot showing the extent of overlap of hits between different CF sputum media and SCFM3. Hits were defined as genes that were statistically significantly enriched or depleted by at least 2-fold in sputum media or SCFM3 compared to TSB. Significance was determined using the resampling method in TRANSIT and *p*-values were adjusted for multiple comparisons using the Benjamini-Hochberg procedure. Hits that were enriched in one or more samples but depleted in one or more other samples were not counted as overlapping. **B)** Heatmap showing all significant genes by ANOVA, with TSB as the reference condition.


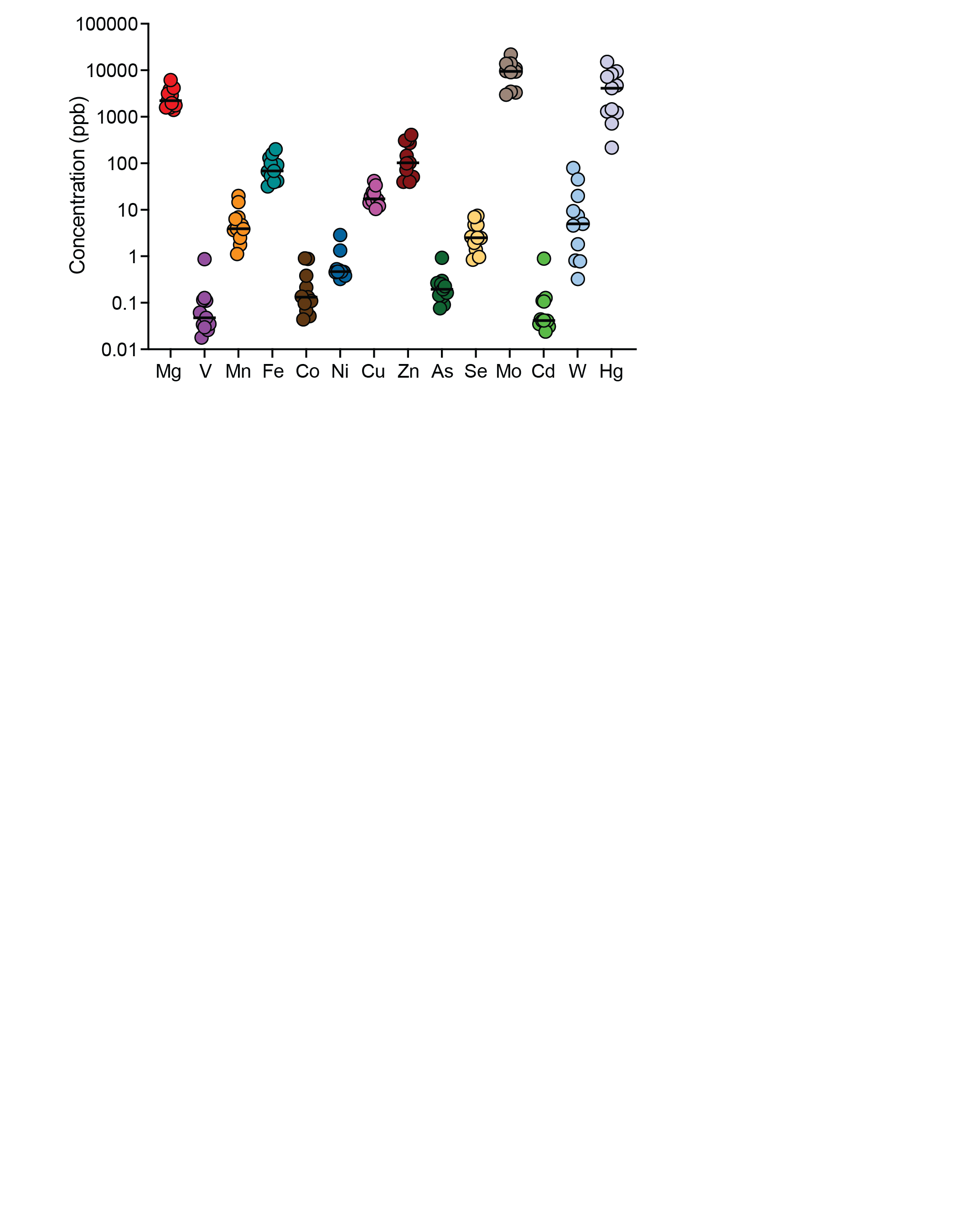


**Figure S2. Elements quantified by ICP-MS in CF sputum.** Each point represents a sputum sample from an individual with CF.


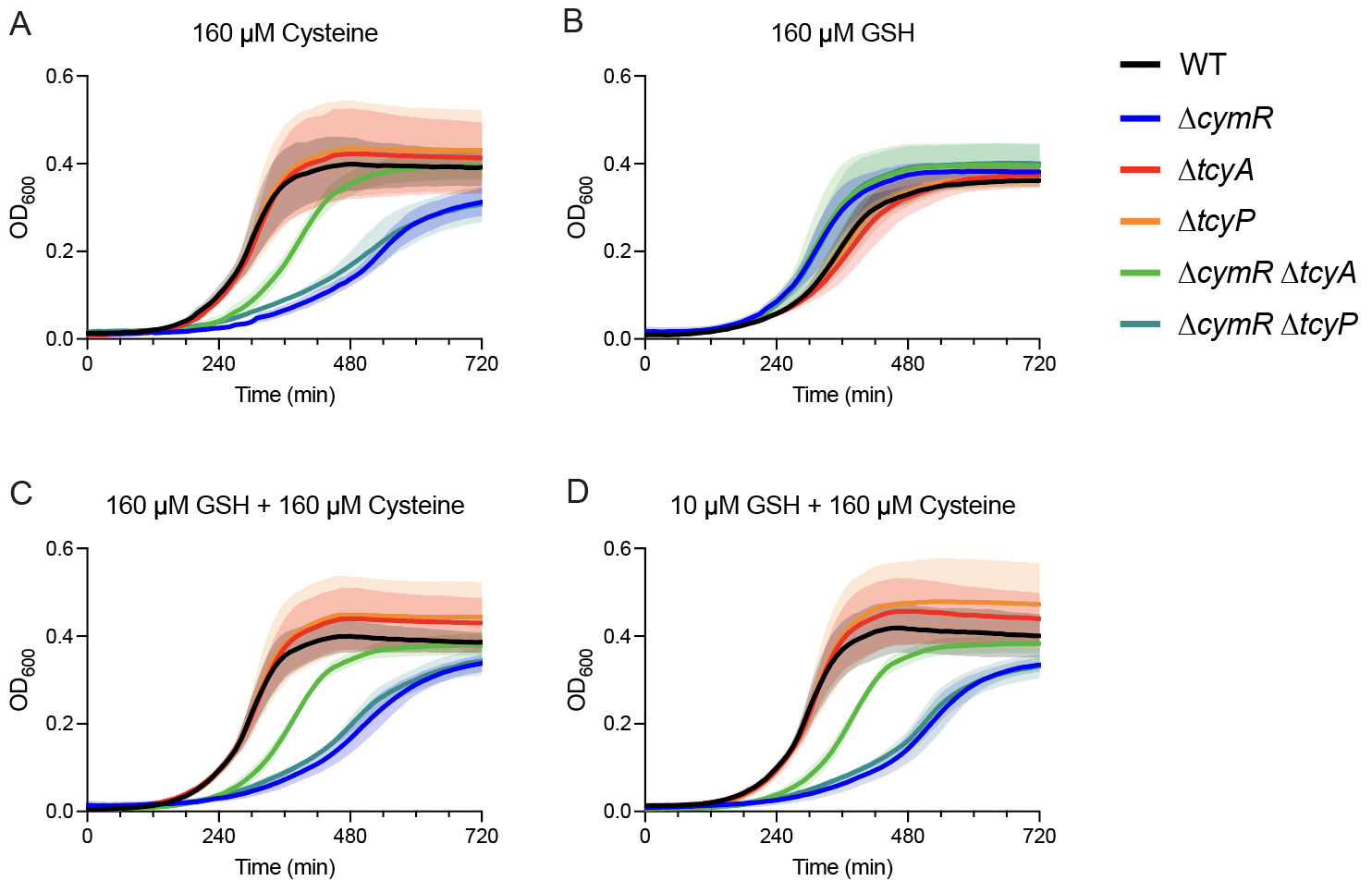


**Figure S3. The role of each cysteine importer in cysteine toxicity of ∆*cymR*.** Growth kinetics of bacterial strains in SCFM1 containing the indicated organic sulfur source: A) cysteine, B) GSH, C) equimolar cysteine and GSH, D) cysteine and minimal GSH. Data are the mean and standard deviation of four independent experiments.

**Table S3.** **Strains used in this study**

| Strain | Source |
| --- | --- |
| *S. aureus* JE2 | 1 |
| *E. coli* IM08B | 2 |
| JE2 ∆*cymR* | This study |
| JE2 ∆*nanE* | This study |
| JE2 ∆*ylaN* | This study |
| JE2 ∆*cntE* | This study |
| JE2 ∆*tcyA* | This study |
| JE2 ∆*tcyP* | This study |
| JE2 ∆*cymR* ∆*tcyA* | This study |
| JE2 ∆*cymR* ∆*tcyP* | This study |
| JE2 ∆*tcyA* ∆*tcyP* | This study |
| JE2 ∆*cymR* ∆*tcyA* ∆*tcyP* | This study |
| JE2 *cntA*::Tn | 1; this study |
| JE2 *mntE*::Tn | 1; this study |
| JE2 *copA*::Tn | 1; this study |
| JE2 *pruA*::Tn | 1; this study |
| JE2 *rpsN*::Tn | 1; this study |
| JE2 ∆*cymR* pOS1-*cymR* | This study |

**Table S4. Plasmids and phages used in this study**

| Plasmid or Phage | Description | Source |
| --- | --- | --- |
| ɸ11::FRT | Generalized transduction phage | 3 |
| pTM239 | *bursa aurealis* blunt donor | 3 |
| pTM241 | *bursa aurealis P*_cap_ donor | 3 |
| pTM242 | *bursa aurealis P*_tuf_ donor | 3 |
| pTM243 | *bursa aurealis P*_erm_ donor | 3 |
| pTM244 | *bursa aurealis P*_dual_ donor | 3 |
| pORF5-Tnp+ | Transposase-expressing plasmid | 3 |
| pORF5-Tnp- | Transposase-negative control | 3 |
| pOS1 | Complementation vector | 4 |
| pIMAY* | Allelic exchange vector | 5 |
| pMUTIN4 | *lacZ* donor | 6 |
| pIMAY*-Z | Allelic exchange vector including *lacZ* | This study |

**Table S5.** **Oligonucleotides used in this study**

| Name | Sequence (5’-3’) | Description | Source |
| --- | --- | --- | --- |
| LS000 | CAAGGTCATAAAGCTTCATTTG | TM58 | 3 |
| LS001 | GATTGAATCGCCTTATGCATG | TM59 | 3 |
| LS002 | CAAGCAGAAGACGGCATACGAAGACC | TM196 | 3 |
| LS003 | CGCTCACCCAAATATATATCTTGATG | TM198 | 3 |
| LS082 | /5biotinTEG/CGTTAGTAACCTTGCGATGTCGATTCACGTTG | TM214 corrected | 3; this study |
| LS005 | GGCCCAACGTGAATCGACATCGCAAGGT | TM215 | 3 |
| LS006 | TTCCCTACACGACGCTCTTCCGATCTAGTCATGCNN | Barcode 1 top | 3 |
| LS007 | TTCCCTACACGACGCTCTTCCGATCTACGTACTGNN | Barcode 2 top | 3 |
| LS008 | TTCCCTACACGACGCTCTTCCGATCTTGACTGCANN | Barcode 3 top | 3 |
| LS009 | TTCCCTACACGACGCTCTTCCGATCTTCGACGATNN | Barcode 4 top | 3 |
| LS010 | TTCCCTACACGACGCTCTTCCGATCTCTAGCATGNN | Barcode 5 top | 3 |
| LS011 | TTCCCTACACGACGCTCTTCCGATCTGACTGTACNN | Barcode 6 top | 3 |
| LS012 | GCATGACTAGATCGGAAGAGCGTCGTGTAGGGAA | Barcode 1 bottom | 3 |
| LS013 | CAGTACGTAGATCGGAAGAGCGTCGTGTAGGGAA | Barcode 2 bottom | 3 |
| LS014 | TGCAGTCAAGATCGGAAGAGCGTCGTGTAGGGAA | Barcode 3 bottom | 3 |
| LS206 | ATCGTCGAAGATCGGAAGAGCGTCGTGTAGGGAA | Barcode 4 bottom corrected | 3; this study |
| LS016 | CATGCTAGAGATCGGAAGAGCGTCGTGTAGGGAA | Barcode 5 bottom | 3 |
| LS017 | GTACAGTCAGATCGGAAGAGCGTCGTGTAGGGAA | Barcode 6 bottom | 3 |
| LS190 | TTCCCTACACGACGCTCTTCCGATCTTTACCGACNN | Barcode 7 top | 3 |
| LS191 | GTCGGTAAAGATCGGAAGAGCGTCGTGTAGGGAA | Barcode 7 bottom | 3 |
| LS192 | TTCCCTACACGACGCTCTTCCGATCTAGTGACCTNN | Barcode 8 top | 3 |
| LS193 | AGGTCACTAGATCGGAAGAGCGTCGTGTAGGGAA | Barcode 8 bottom | 3 |
| LS194 | TTCCCTACACGACGCTCTTCCGATCTTCGGATTCNN | Barcode 9 top | 3 |
| LS195 | GAATCCGAAGATCGGAAGAGCGTCGTGTAGGGAA | Barcode 9 bottom | 3 |
| LS196 | TTCCCTACACGACGCTCTTCCGATCTCAAGGTACNN | Barcode 10 top | 3 |
| LS197 | GTACCTTGAGATCGGAAGAGCGTCGTGTAGGGAA | Barcode 10 bottom | 3 |
| LS198 | TTCCCTACACGACGCTCTTCCGATCTTCCTCATGNN | Barcode 11 top | 3 |
| LS199 | CTAGAGGAAGATCGGAAGAGCGTCGTGTAGGGAA | Barcode 11 bottom | 3 |
| LS200 | TTCCCTACACGACGCTCTTCCGATCTGTCAGTCANN | Barcode 12 top | 3 |
| LS201 | TGACTGACAGATCGGAAGAGCGTCGTGTAGGGAA | Barcode 12 bottom | 3 |
| LS078 | CAAGCAGAAGACGGCATACGAGATAGACCACGCGTGCCATAAC | Final amplification | This study |
| LS079 | AATGATACGGCGACCACCGAGATCTACACTCTTTCCCTACACGACGCTCTTCCGATCT | TruSeq universal adapter | Illumina |
| LS020 | CTGTCCGTTCCGACTACCCTCCCGAC | M12T | 7 |
| LS021 | GTCGGGAGGGTAGTCGGAACGGACAG | M12B | 7 |
| LS026 | CAGATAGGCCTAATGACTGGCTTTTATAAAGGTGGTGAACTACTGTGGAAGTTACTGACG | Amplify *lacZ* for pIMAY*-Z | This study |
| LS027 | GTAAAAAGTACAGTCGGCATTATCTCATATTATTTTTGACACCAGACCAACTGGTAATGG | Amplify *lacZ* for pIMAY*-Z | This study |
| LS028 | TTATAAAAGCCAGTCATTAGGCCTATCTGAC | Amplify pIMAY* to insert *lacZ* | This study |
| LS029 | TATGAGATAATGCCGACTGTACTTTTTACAG | Amplify pIMAY* to insert *lacZ* | This study |
| LS238 | CCGCTCTAGAACTAGTGGATCCCCCGGTAATCGATTTAATCTTATTTTTACGGCC | *cymR* deletion F1 | This study |
| LS239 | GTATAGTATTTATACGATTACATTGTATAACCTCACTTAATTCGAATATTGATATTCCC | *cymR* deletion R1 | This study |
| LS240 | GAATTAAGTGAGGTTATACAATGTAATCGTATAAATACTATACTATAACATAAAAACTTC | *cymR* deletion F2 | This study |
| LS241 | GCTTGATATCGAATTCCTGCAGCCCGCTCACCAACAAGCATATGTTCGTGC | *cymR* deletion R2 | This study |
| LS250 | GACTGGAATTCTCTTCTGATAATGCCTCTTGGTCC | *cymR* complement | This study |
| LS251 | CAGTCGGATCCTTAAATATAAAACATGTATCCGTC | *cymR* complement | This study |
| LS244 | CCGCTCTAGAACTAGTGGATCCCCCATTTATAGATATTAAAAAACACTTTGACTGTGCG | *cntE* deletion F1 | This study |
| LS245 | CACTTCATTACATTATCTCACTCCTTAGAATGATAGTTGTTTTTCTATTAATTCTCTTG | *cntE* deletion R1 | This study |
| LS246 | CTATCATTCTAAGGAGTGAGATAATGTAATGAAGTGAATTAAAGCATATTAAGTTAATG | *cntE* deletion F2 | This study |
| LS247 | GCTTGATATCGAATTCCTGCAGCCCTATATAATACAACTATTTAAGAGGTATACTATGTC | *cntE* deletion R2 | This study |
| LS316 | CCGCTCTAGAACTAGTGGATCCCCCTTGCTTAGCTTCTCTTTTGGCATATAATATTTG | *ylaN* deletion F1 | This study |
| LS317 | GCTTATATATTAAACCTTACATGACACGCTCCCCCTCTATATAAATTCTCAAGTC | *ylaN* deletion R1 | This study |
| LS318 | GAGGGGGAGCGTGTCATGTAAGGTTTAATATATAAGCATATTAAAATGGCGTGAG | *ylaN* deletion F2 | This study |
| LS319 | GCTTGATATCGAATTCCTGCAGCCCGGACTTTGTTTACCCCAATTCCTG | *ylaN* deletion R2 | This study |
| LS336 | CCGCTCTAGAACTAGTGGATCCCCCCTACAATTAAAGTACCTATTGATTTTATTTCTGTC | *tcyA* deletion F1 | This study |
| LS337 | CCAAACCATTTCTTACCTATCATAGAATATATCTCCTTATTCTTATTATTCTAATCGG | *tcyA* deletion R1 | This study |
| LS338 | GGAGATATATTCTATGATAGGTAAGAAATGGTTTGGTCAAGATGTTTCTAAATC | *tcyA* deletion F2 | This study |
| LS339 | GCTTGATATCGAATTCCTGCAGCCCCTTTGAAATACCATACCAGACTGTTTACG | *tcyA* deletion R2 | This study |
| LS342 | CCGCTCTAGAACTAGTGGATCCCCCGCACGTTTATTCAACCTGTAAACTTGTGATTTG | *tcyP* deletion F1 | This study |
| LS343 | CGGGTATTTCTTGCAATGAATTACATGAACGTCACTCCTCAAATTTTTGAATATAATTCC | *tcyP* deletion R1 | This study |
| LS344 | GGAGTGACGTTCATGTAATTCATTGCAAGAAATACCCGCCCCCTCTAGCTATACTTATC | *tcyP* deletion F2 | This study |
| LS345 | GCTTGATATCGAATTCCTGCAGCCCGAAGGTAATGAGTGAGAAACTGGATTTTTAAAG | *tcyP* deletion R2 | This study |
| LS354 | CCGCTCTAGAACTAGTGGATCCCCCGCACCTAGCAACTCGTTGGGACAATCACGATG | *nanE* deletion F1 | This study |
| LS355 | CTCGTTTTTTTATCGTTATCATTTACATTATTCCTCACTCCATAAGTCGTTTTTTCACG | *nanE* deletion R1 | This study |
| LS356 | GTGAGGAATAATGTAAATGATAACGATAAAAAAACGAGATGACCATCATTAATTAAAGG | *nanE* deletion F2 | This study |
| LS357 | GCTTGATATCGAATTCCTGCAGCCCCCGCTGTCGATGCAGTGGCTTCGTTTGTCGG | *nanE* deletion R2 | This study |

**SUPPLEMENTAL REFERENCES**

1. Fey, P. D. *et al.* A genetic resource for rapid and comprehensive phenotype screening of nonessential Staphylococcus aureus genes. *mBio* **4**, e00537-00512 (2013).

2. Monk, I. R., Tree, J. J., Howden, B. P., Stinear, T. P. & Foster, T. J. Complete Bypass of Restriction Systems for Major Staphylococcus aureus Lineages. *mBio* **6**, e00308-00315 (2015).

3. Santiago, M. *et al.* A new platform for ultra-high density Staphylococcus aureus transposon libraries. *BMC Genomics* **16**, 252 (2015).

4. Schneewind, O., Model, P. & Fischetti, V. A. Sorting of protein A to the staphylococcal cell wall. *Cell* **70**, 267–281 (1992).

5. Schuster, C. F., Howard, S. A. & Gründling, A. 2019. Use of the counter selectable marker PheS* for genome engineering in Staphylococcus aureus. *Microbiology* **165**, 572–584 (2019).

6. Vagner, V., Dervyn, E. & Ehrlich, S. D. A vector for systematic gene inactivation in Bacillus subtilis. *Microbiol. Read. Engl.* **144 (Pt 11)**, 3097–3104 (1998).

7. Morgan, R. D., Bhatia, T. K., Lovasco, L. & Davis, T. B. MmeI: a minimal Type II restriction-modification system that only modifies one DNA strand for host protection. *Nucleic Acids Res.* **36**, 6558–6570 (2008).
